# Brain activity mediates the relation between emotional but not instrumental support and trait loneliness

**DOI:** 10.1093/scan/nsy067

**Published:** 2018-08-24

**Authors:** Yangyang Yi, Liman Man Wai Li, Yu Xiao, Junji Ma, Linlin Fan, Zhengjia Dai

**Affiliations:** 1 Department of Psychology, Sun Yat-sen University, Guangzhou, China; 2 Department of Psychology and Centre for Psychosocial Health, The Education University of Hong Kong, Hong Kong SAR, China

**Keywords:** loneliness, trait loneliness, emotional support, instrumental support, inferior temporal gyrus, resting-state fMRI

## Abstract

Loneliness results from lacking satisfied social connections. However, little is known how trait loneliness, which is a stable personal characteristic, is influenced by different types of social support (i.e. emotional and instrumental support) through the brain activity associated with loneliness. To explore these questions, data of resting-state functional magnetic resonance imaging (R-fMRI) of 92 healthy participants were analyzed. We identified loneliness-related brain regions by correlating participants’ loneliness scores with amplitudes of low-frequency fluctuation (ALFF) of R-fMRI data. We then conducted mediation analyses to test whether the negative relation between each type of social support and loneliness was explained via the neural activity in the loneliness-related brain regions. The results showed that loneliness was positively related to the mean ALFF value within right inferior temporal gyrus (ITG). In addition, the negative relation between emotional support and loneliness was explained by a decrease in the spontaneous neural activity within right ITG but this pattern was not observed for instrumental support. These results suggest the importance of social information processing on trait loneliness and highlight the need to differentiate the functions of different types of social support on mental health from a neural perspective.

## Introduction

Loneliness, which results from subjective perception of deficiencies in intimate social relationships (Peplau and Perlman, 1982; Cacioppo and Patrick, 2008), is increasingly prevalent in the modern society. Numerous studies have demonstrated that loneliness is an important risk factor not only for mental health (e.g. depression and schizophrenia) (Richman and Sokolove, 1992; Deniro, 1995; Bunney *et al.*, 2002; Cacioppo *et al.*, 2006) but also for morbidity and mortality (Berkman and Syme, 1979; Cacioppo *et al.*, 2006; Shiovitz-Ezra and Ayalon, 2010; Steptoe *et al.*, 2013; Holt-Lunstad *et al.*, 2015). Previous work showed that loneliness can be situationally induced (i.e. state loneliness) or a dispositional condition (i.e. trait loneliness), and these two types of loneliness were found not to be strongly correlated (Cairns *et al.*, 1995). Importantly, trait loneliness is suggested to be more detrimental than state loneliness, in which trait loneliness is more likely to be associated with passive coping strategies such as drug abuse (Shaver *et al.*, 1985) and its effect is persistent regardless of situational factors, making people feel lonelier across situations (Cheek and Busch, 1981).

Given the detrimental influences of trait loneliness on both physical and mental health, it is important to develop interventions to reduce it. Social relationship experiences and activities were found to underlie people’s loneliness level (Carmona *et al.*, 2014); thus interventions that help correct social deficits in various domains of social interactions, including social support, have been developed (for a review, see Masi *et al.*, 2011). However, the literature reports mixed results on the beneficial effects of social support on loneliness (Masi *et al.*, 2011), with some studies reporting that social support could effectively reduce loneliness (Collins and Benedict, 2006; Ollonqvist *et al.*, 2008; Salsman *et al.*, 2013), and other studies reporting that social support did not have any impact on loneliness (Heller *et al.*, 1991; Coleman *et al.*, 2005; Winningham and Pike, 2007). The present study proposes that a careful distinction between emotional support and instrumental support, which has been often disregarded in previous studies (Malecki and Demaray, 2003), may provide insights for solving the mixed results. Evidence converges to show that these two types of social support do not lead to identical effects (Barrera, 1986; House *et al.*, 1988; Bolger *et al.*, 2000); thus it is important to further pinpoint what type of social support would be more effective in reducing loneliness.

Emotional support refers to caring and understanding from others and intimate sharing with others; while instrumental support refers to material or functional aids in daily tasks from others (Langford *et al.*, 1997). Compared with instrumental support, emotional support is suggested to be more likely to help facilitate intimate sharing with others (Chen and Silverstein, 2000) and reduce unsatisfied feeling in a relationship (Reis *et al.*, 2004; Maisel and Gable, 2009), which may in turn reduce loneliness resulting from a lack of intimacy in social experiences (Baumeister and Leary, 1995; Cacioppo and Patrick, 2008). The functions of these two types of social support suggest that emotional support would be more beneficial in reducing loneliness. Consistent with this speculation, Sorkin *et al*. (2002) contended that unmet needs for emotional support are one of the important underlying social deficits that cause loneliness. In addition, evidence obtained in previous research on other well-being indicators that are highly related to loneliness provides insights to our research question. Some studies discovered that emotional support had stronger influences on well-being than had instrumental support (Rook, 1987; Krause and Liang, 1993; Liu *et al.*, 1995; Hombrados-Mendieta *et al.*, 2013); while some studies even found that emotional but not instrumental support predicted better psychological well-being (Kaufmann and Beehr, 1989; Adams *et al.*, 1996). Given the importance of unmet needs for emotional support for giving rise to loneliness (Sorkin *et al.*, 2002) and supportive evidence from the research on other well-being indicators, we hypothesize that a stronger negative relation would be observed between emotional support and loneliness than between instrumental support and loneliness.

The brain is the key organ creating, monitoring and maintaining people’s feelings during social interactions (Cacioppo *et al.*, 2015). Therefore, understanding the neural mechanisms of the relation between social support and loneliness is crucial to advance the understanding of loneliness (for review, see Cacioppo *et al.*, 2015). However, to our knowledge, no studies have examined how the neuroimaging biomarkers, which are suggested to be observed before the onset of different types of disorders (Joormann *et al.*, 2012; Hosseini *et al.*, 2014) and are important for evaluating the effectiveness of interventions or psychotherapies (Klumpp *et al.*, 2017), link social support to loneliness.

To examine the neural mechanisms for the relation between social support and loneliness, following the methodology used in previous clinical research (Ecker *et al.*, 2010; Zeng *et al.*, 2012), we first needed to identify the brain regions related to loneliness. However, previous task-based functional magnetic resonance imaging (fMRI) studies did not provide a conclusive answer. Cacioppo *et al.* (2009) found that lonely participants showed weaker neural activities in the ventral striatum and bilateral temporoparietal junction when viewing the picture of people *vs* objects, while this pattern was reversed for non-lonely participants. Inagaki *et al.* (2015) found that loneliness level was positively related to an increase in ventral striatum activity when participants viewed a close other *vs* a stranger. Wong *et al.* (2016) found that loneliness level was positively related to functional activity within frontal lobe and subcortical regions when participants viewed affective stimuli. Some studies also found that, when confronted with social exclusion or separation from caregivers, which was expected to induce loneliness, neural activity in inferior temporal gyrus (ITG) was increased (Rilling *et al.*, 2001; Bolling *et al.*, 2011). To summarize, the previous findings discovered dispersive loneliness-related brain regions.

All mentioned fMRI studies adopted a task-based approach, which is situation-dependent, revealing the neural reaction of lonely *vs* non-lonely people to different types of stimuli or how people respond to loneliness-induced situations (i.e. state loneliness). Resting-state fMRI (R-fMRI), without using any explicit tasks, explores the spontaneous neural activity of the brain (Biswal *et al.*, 1995), which reflects people’s previous learning experiences (Tavor *et al.*, 2016) and stable behavioral tendencies (Cole *et al.*, 2012). Therefore, it is no surprise that R-fMRI has been used to explore the neural bases of individual differences in a variety of stable personal characteristics, such as personality (Canli, 2004; Kennis *et al.*, 2013), well-being (Kong *et al.*, 2015), trait anxiety (Tian *et al.*, 2016) and self-construal (Li *et al.*, 2018). Therefore, it may be more appropriate to use R-fMRI to identify the neural mechanisms associated with trait loneliness, which is relatively stable across time points. However, little has been done previously.

In the present study, we first examined whether emotional support and instrumental support would be negatively related to loneliness, respectively. Next, considering the dispersive loneliness-related brain regions found in the previous task-based fMRI studies and the differences between state loneliness and trait loneliness, we first explored loneliness-related regions with R-fMRI data. To avoid missing any brain regions that are related to loneliness, we conducted a whole-brain voxel-based analysis by correlating the amplitude of low-frequency fluctuation (ALFF) (Zang *et al.*, 2007) values of every voxel in the gray matter (GM) with individuals’ loneliness scores in 92 healthy participants to identify the brain regions related to loneliness. The ALFF index can capture the regional intensity of spontaneous or intrinsic fluctuations in blood oxygenation level-dependent (BOLD) signals and reflect the regional metabolic level of glucose (Tomasi *et al.*, 2013). This index has been used to detect neuroimaging biomarkers underlying different neuropsychiatric illnesses like anxiety (e.g. Wang *et al.*, 2017) and individual differences like well-being (e.g. Kong *et al.*, 2015). Finally, we explored whether the negative relation between social support and loneliness could be explained via the spontaneous neural activity within loneliness-related brain regions.

## Materials and methods

### Participants

Data from 100 healthy adults (the ‘Unrelated 100’ group from the released database of 900 participants; all participants were between 22 and 35, except one participant was over 36 years old; 54 females) were obtained from Human Connectome Project database. This project was supported by the WU-Minn Consortium (Van Essen *et al.*, 2013). Each participant had written the informed consent. This research was approved by the local institutional review board at Washington University in St. Louis.

### Behavioral data acquisition

All participants finished scales measuring their loneliness level, perceived emotional support and perceived instrumental support (Salsman *et al.*, 2013) from NIH Toolbox (http://www.nihtoolbox.org). The loneliness scale measured how often participants perceived alone, lonely or socially isolated in the past month with five items (e.g. ‘I feel alone and apart from others’). Emotional support scale measured how often participants perceived that there was someone available to listen to their problems with empathy, carefulness and understanding in the past month with eight items (e.g. ‘I feel there are people I can talk to if I am upset’). Instrumental support scale measured how often participants perceived that they could get practical or material support when needed in the past month with eight items (e.g. ‘I have someone to take me to the doctor if I need it’). Participants indicated their responses with a 5-point scale (1 = *never* and 5 = *always*) for all measures. The final score of each scale was converted to a theta score with the mean of 50 and the s.d. of 10 based on item response theory (IRT; for details, see http://www.nihtoolbox.org/HowDoI/Pages/ScoringAndInterpretation.aspx).

### R-fMRI data acquisition

Every participant was scanned with two sessions, and each session included two runs. One run was phase encoded from left to right and the other was phase encoded from right to left. The left-to-right encoding dataset of the first session was used for the main analysis and the right-to-left encoding dataset of the first session was used for the validation analysis.

All participants were scanned in a 32-channel Siemens 3T ‘Connectome Skyra’ scanner. Functional images were collected using a multiband gradient-echo-planar imaging sequence. The image parameters were as follows: time repetition = 720 ms; time echo = 33.1 ms; flip angle = 52°; field of view = 208 × 180 mm^2^; matrix = 104 × 90; slices number = 72; slice thickness = 2 mm; voxel size = 2 × 2 × 2 mm^3^; multiband factor = 8; and 1200 volumes. Participants were asked to keep their eyes open, fixate on a bright cross-hair presented on a black background and relax.

### Image pre-processing

The R-fMRI data have been pre-processed with minimal pre-processing pipeline which included artifact removal, motion correction and registration to standard space (Glasser *et al.*, 2013). Seven participants with > 0.14 mm mean frame-to-framehead motion estimate were excluded from further analyses (Finn *et al.*, 2015; Liao *et al.*, 2017). Considering that age has a significant influence on the structure and function of the brain (Raz, 2000), one participant whose age was unknown (36+) was also excluded from further analyses, leaving 92 participants (age between 22 and 35, 52 females). The data including this participant were also analyzed and the results were substantively unchanged (for details, see Supplementary Information). Additional pre-processing steps were conducted to reduce artificial signals or physiological noise with a MATLAB toolbox called Data Processing & Analysis for Resting-State Brain Imaging (DPABI) (Yan *et al.*, 2016). These steps included (i) smoothing with a 4 mm full-width half-maximum Gaussian kernel, (ii) removing the systematic trend, and (iii) regressing out Friston 24-parameters (Friston *et al.*, 1996), cerebrospinal fluid (CSF) signals, whitematter (WM) signals and global signals.

### Defining loneliness-related brain regions

The amplitude of regional spontaneous neural activity was presented with ALFF (Zang *et al.*, 2007). Briefly, we first converted the time series of every voxel to the frequency domain using fast Fourier transformation. The square root of the power spectrum was computed and then averaged across 0.01–0.08 Hz, which was termed the ALFF. Higher ALFF reflects greater neural activity.

We first correlated the participants’ loneliness scores with ALFF values in a voxel-wise manner within GM mask, which was generated by thresholding (0.2) a priori GM probability map in Statistical Parametric Mapping (SPM8, http://www.fil.ion.ucl.ac.uk/spm). The statistical significance threshold was set at *P* < 0.001 and cluster size was greater than 60 voxels, which corresponded to a corrected *P* < 0.05. This correction was performed by Monte Carlo simulation using the r-fMRI data analysis toolkit (REST toolkit) (Song *et al.*, 2011). We defined brain regions whose ALFF values were significantly correlated with loneliness score as loneliness-related regions. The ALFF values within loneliness-related regions were then averaged as the indicator of the amplitude of neural activity within loneliness-related regions.

### The relations among social support, loneliness, and loneliness-related brain regions

Emotional support and instrumental support were significantly correlated with each other (Semmer *et al.*, 2008; Morelli *et al.*, 2015). To explore the unique effect of each type of social support on loneliness, two separate general linear regression analyses were conducted with emotional support (or instrumental support) as the independent variable with controlling for the effect of instrumental support (or emotional support), and loneliness as the dependent variable, respectively. In addition, prior work revealed stable gender differences in responses to emotional and instrumental support (Day and Livingstone, 2003); thus gender was also controlled.

To examine whether emotional and instrumental support, separately, would be negatively related to loneliness via the spontaneous neural activity within loneliness-related regions. Two mediation analyses were conducted with emotional support (or instrumental support) as the independent variable with controlling for the effect of instrumental support (or emotional support) and gender, the mean ALFF value within the loneliness-related regions as the mediator and loneliness as the dependent variable following the procedures developed by Preacher and Hayes (2008). To test whether these mediation models were significant, the analyses were conducted with 5000 bootstrap samples and bias-corrected confidence intervals (CI) were calculated.

## Results

Table 1 presents the means, standard deviations (s.d.) of loneliness, emotional and instrumental support scores and the inter-correlations among them.

**Table 1:** Descriptive statistics and inter-correlations among the key variables

	Mean (s.d.)	1	2	3
1. Loneliness score	50.531 (8.631)	−		
2. Emotional support score	51.770 (9.475)	−0.655^***^	−	
3. Instrumental support score	49.953 (8.226)	−0.466^***^	0.481^***^	−

*** ^***^ *P* < 0.001

### Relations between two types of social support and loneliness

Our results showed that emotional support was negatively correlated with loneliness with controlling for the effects of instrumental support and gender, *b* = −0.540, *P* < 0.001, 95% CI = (−0.706, −0.374), while instrumental support was not significantly correlated with loneliness with controlling for the effects of emotional support and gender, *b* = −0.169, *P* = 0.082, 95% CI = (−0.360, 0.022).

### Loneliness-related brain regions

**Fig. 1:**
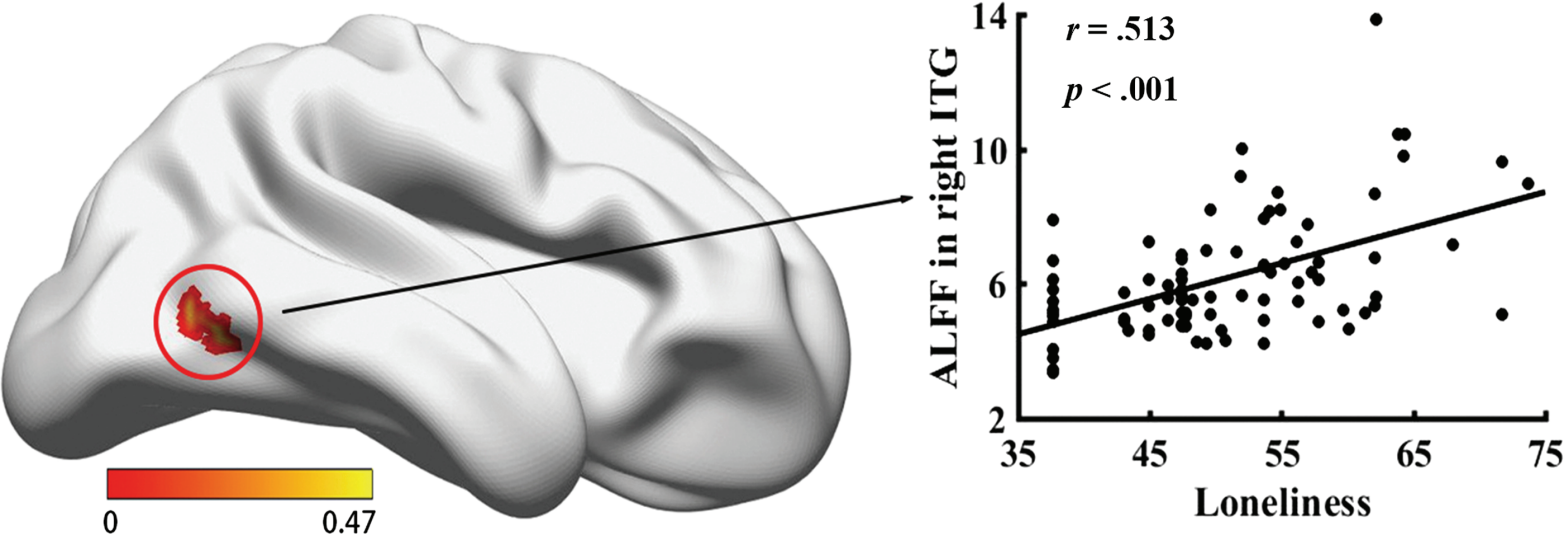
Loneliness-related brain region. Brighter color indicates higher correlation coefficients (**A**). The relationship between mean ALFF within the loneliness-related region (inferior temporal gyrus) and loneliness (**B**). The mean neural activity in this region was positively correlated with loneliness scores. The visualization was provided by with BrainNet Viewer (http://www.nitrc.org/projects/bnv/).

Only one cluster survived with the adopted threshold, which located in the right ITG (MNI of peak voxel: [52, −62, −12] mm; Figure 1A), and we defined this cluster as the loneliness-related region. Mean ALFF value of this cluster was positively correlated with loneliness, *r* = 0.513, *P* < 0.001 (Figure 1B). We have identified one participant with a mean ALFF value within right ITG above 3 s.d. Although the results remained similar after excluding the data of this participant (*r* = 0.512, *P* < 0.001), we reported the analyses on the data that excluded this participant in the main text to exclude the possibility that the obtained results were driven by this specific participant. To advocate for the full transparency of analyses, we re-analyzed the data including this outlier and the results were essentially unchanged (for details, see Supplementary Information).

### Neural activity within loneliness-related brain region mediated the relation between emotional support (but not instrumental support) and loneliness

**Fig. 2:**
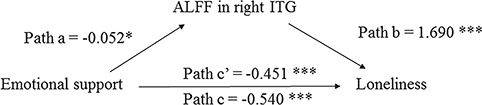
Mediation analysis. The relation between emotional support and loneliness scores was mediated by the mean ALFF value within the loneliness-related region (ITG). Unstandardized regression coefficients were reported. ^*^P < 0.05, ^***^P < 0.001.

Regarding the influence of emotional support, the results showed that greater perceived emotional support was associated with lower loneliness, *b =* −0.540, *P* < 0.001. In addition, greater perceived emotional support was associated with a lower mean ALFF value within right ITG, *b =* −0.052, *P* = 0.011. Furthermore, a lower mean ALFF value within right ITG was associated with lower loneliness, *b* = 1.690, *P* < 0.001. After considering the effect of the mean ALFF value within right ITG, the relation between emotional support and loneliness was weakened, *b* = −0.451, *P* < 0.001 (from *b =* −0.540, *P* < 0.001). More importantly, the mediation analysis indicated that the mean ALFF value within right ITG was a significant mediator, 95% CI = (−0.225, −0.019), partially explaining the negative relation between emotional support and loneliness (Figure 2).

**Fig. 3:**
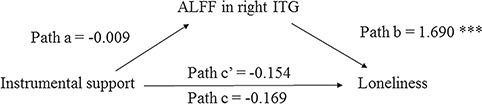
Mediation analysis. The relation between instrumental support and loneliness scores was mediated by the mean ALFF value within the loneliness-related region (ITG). Unstandardized regression coefficients are reported. ^***^P < 0.001.

Regarding the influence of instrumental support, the results showed that perceived instrumental support was not associated with loneliness, *b =* −0.169, *P* = 0.082, and the mean ALFF value within right ITG, *b =* −0.009, *P =* 0.696, although a lower mean ALFF value within right ITG was associated with lower loneliness, *b* = 1.690, *P* < 0.001. After considering the effect of the mean ALFF value within right ITG, the relation between instrumental support and loneliness remained non-significant, *b* = −0.154, *P* = 0.086. The mediation analysis indicated that the mean ALFF value within right ITG was not a significant mediator, 95% CI = (−0.107, 0.054) (Figure 3).

### Replication results across datasets

In order to ensure that our results can be reliably reproduced, we replicated our analyses in another dataset (i.e. the run with right-to-left encoding in the same session). The results showed that spontaneous neural activity in right ITG was positively correlated with loneliness, *r* = 0.386, *P* < 0.001. We identified two participants with mean ALFF values within right ITG above 3 s.d. Although the results remained similar after excluding the data of these two participants (*r* = 0.413, *P* < 0.001), we excluded these two participants in following analyses, which was consistent with our main analysis. The mediation results showed that the mean ALFF value in right ITG mediated the relation between emotional support and loneliness, 95% CI = (−0.139, −0.001), but it did not mediate the relation between instrumental support and loneliness, 95% CI = (−0.103, 0.017). Taken together, all results were replicated in this independent dataset.

## Discussion

To examine the neural bases of how emotional support and instrumental support relate to loneliness, we conducted an ALFF-behavior analysis with R-fMRI data. The results showed that the spontaneous neural activity within right ITG was positively related to loneliness, meaning that lonely individuals had greater spontaneous neural activity in right ITG. Furthermore, emotional but not instrumental support was negatively related to loneliness, which was explained by a decrease in the neural activity within right ITG.

### Neural bases of loneliness

The current findings showed that right ITG was an important brain region related to loneliness. In addition to its involvement in basic cognitive processes, such as shape processing (Creem and Proffitt, 2001; Denys *et al.*, 2004), ITG was also identified to be an important brain region that involved in processing of social information, such as face recognition and emotion recognition (Haxby *et al.*, 2001; Rossion *et al.*, 2003; Barton *et al.*, 2004). Lonely people reported a higher level of need to belong (Peplau and Perlman, 1982; DeWall and Richman, 2011), which could promote greater attention to social cues, such as vocal tone and facial emotion of others (Pickett *et al.*, 2004). The positive relation between loneliness and spontaneous activity in ITG, which is one of brain regions that participates in social-information processes, may reflect the greater readiness of processing social information among lonely people. Our results were consistent with the previous studies on loneliness-related constructs. For example, social isolation, which is closely related to loneliness (Weiss, 1973), was found to be positively correlated with neural activity in ITG (Rilling *et al.*, 2001; Bolling *et al.*, 2011). In an animal study, isolation from caregivers was found to induce more intense neural activity in ITG (Rilling *et al.*, 2001). Depressive symptom, which is often concurrent with higher loneliness (Cacioppo *et al.*, 2006), was related with increased functional connectivity density in ITG (Lan *et al.*, 2015) and increased regional cerebral blood flow in ITG (Van Heeringen *et al.*, 2010). These findings converge to suggest that the neural activity of ITG may be a potential neuroimaging biomarker of loneliness.

However, besides ITG, the current study failed to identify other brain regions that were found to be correlated with loneliness in previous work (e.g. frontal lobe, temporoparietal junction and subcortical regions). One possibility for the discrepancy is that the previous studies used task-based fMRI, so the identified regions could be specific to the stimuli or situations involved in the studies. In other words, the role of the unidentified brain areas may be more salient when people process loneliness-related content (i.e. state loneliness). The current findings were also not consistent with a recent study examining neural functional connectivity related to social isolation, a concept that is closely related to loneliness (but conceptually distinctive), with R-fMRI data (Layden *et al.*, 2017). They found that social isolation was positively correlated with functional connectivity strength of right central operculum and right supramarginal gyrus, which are associated with attentional processes. These inconsistent findings can be due to the differences in the neural indices used (i.e. functional connectivity *vs* ALFF), which emphasize different aspects of neural mechanisms, and the differences in the concepts tested (i.e. social isolation *vs* loneliness) (Weiss, 1973). In addition, it may be also due to the fact that loneliness is a complex social emotion, which is associated with not only changed attentional processes, the focus of the Layden *et al.*’s work, but also changed social and emotional processes (Cacioppo and Hawkley, 2009). Future research should combine multi-modal neuroimaging data (e.g. MRI, diffusion MRI and fMRI) and multiple measures (e.g. GM volume, ALFF and functional connectivity) (Dai *et al.*, 2012) to comprehensively understand the neural bases of loneliness.

### The relations between two types of social support and loneliness

One highlight of the current study was that we investigated the relations between different types of social support and loneliness from a neural perspective. The obtained findings may bring some insights into the research of social support. First, the results provided further support for the beneficial role of emotional support in reducing loneliness, which was consistent with previous work (Ellwardt *et al.*, 2013; Morelli *et al.*, 2015). In contrast, no notable evidence in the current research supported the beneficial role of instrumental support while prior work obtained inconsistent patterns (Ellwardt *et al.*, 2013; Hombrados-Mendieta *et al.*, 2013). Considering the mixed results, future research should identify the boundary conditions of when instrumental support would be protective, which is crucial for reconciling the inconsistent results.

More importantly, the current study is the first study revealing that emotional but not instrumental support was correlated with the neural activity of the brain region related to loneliness (i.e. ITG), which is not only consistent with behavioral research in social psychology research but also potentially crucial for advancing the neuroscience research in social support and loneliness. Emotional support can promote a sense of belonging to social networks (Cobb, 1976), which can facilitate good social interactions (Hagerty *et al.*, 1996; Juvonen, 2006) that allow people to improve their ability of social information processing, which may be finally reflected in the neural function in ITG (Wicker *et al.*, 1998; Barton *et al.*, 2004).

Furthermore, the present study may have some practical implications. As loneliness is highly associated with a lot of mental disorders like depression, phobia and obsession (Meltzer *et al.*, 2013)**,** a better understanding of the neural mechanisms of the trait component of loneliness can provide some insights in searching a way to alert people’s chronic loneliness level, which can potentially promote better well-being. Previous work found that repeatedly exposing to the same environment or condition, like repeatedly engaging in cognitive therapy and meditation, can shape the neural mechanisms underlying different social and emotional behaviors (for review, see Davidson and McEwen, 2012), and in turn, result in better well-being. Considering the current findings, we speculate that living in an environment with continuous emotional support may shape the intrinsic neural function in right ITG, which may help reduce individuals’ loneliness level. Future research should conduct a longitudinal study to test this possibility.

### Limitations and further directions

There were some limitations in the present study. First, following the methodology of previous research on psychotherapies (e.g. Klumpp *et al.*, 2017), we tested whether social support could influence the intrinsic neural activity in loneliness-related brain regions, which could in turn influence the level of trait loneliness. However, all of our analyses were correlational, which did not allow us to examine the causal relations among social support, spontaneous neural activity in right ITG and loneliness. To address this concern, future studies can render social support to observe whether the change in emotional support would cause a decline in neural activity within right ITG and in turn lead to a lower level of loneliness. Second, the age range of participants in our study was rather restricted (between 22 and 35 years old), which may limit the generalizability of the obtained results (Ellwardt *et al.,*^2013^). Further work should investigate whether age would moderate the effect of social support on loneliness from both behavioral and neural perspectives. Third, although the self-report method is an effective method to resolve many psychological problems, it cannot avoid some limitations (e.g. social desirability bias and demand characteristics), especially when the examined concepts are personally sensitive. Future research should adopt behavioral or implicit measures for social support and loneliness. Finally, our work primarily focused on identifying the intrinsic brain activity associated with the trait component of loneliness. One important question that how people varying in the level of chronic loneliness respond to lonely situations was not explored systematically. Future work can consider combining R-fMRI and task-based fMRI to fully explore how chronically lonely *vs* non-lonely people would respond to different situations, which will help comprehend the understanding of the neural mechanisms of both the trait and state component of loneliness.

In conclusion, the present study showed that loneliness was associated with greater spontaneous neural activity in right ITG, and the negative relation between emotional support and loneliness was partially through reducing the neural activity in right ITG. In contrast, we did not find reliable evidence for the relation between instrumental support and loneliness. The present study advanced the understanding of loneliness and social support, which are both important to mental and physical health status, from a neural perspective.

*Conflict of interest*. None declared.

## Funding

This work was supported by the National Natural Science Foundation of China (81601559 and 71701219), Guangdong Provincial Natural Science Foundation of China (2016A030310233) and the Humanities and Social Sciences Foundation of the Ministry of Education of China (16YJC190011).
